# Draft genome of *Staphylococcus capitis* O112, isolated from the cheek swab of a healthy female

**DOI:** 10.1128/mra.01271-23

**Published:** 2024-02-09

**Authors:** Sandra Jablonska, Alex Kula, Catherine Putonti

**Affiliations:** 1 Department of Biology, Loyola University Chicago, Chicago, Illinois, USA; 2 Bioinformatics Program, Loyola University Chicago, Chicago, Illinois, USA; Portland State University, USA

**Keywords:** *Staphylococcus capitis*, oral microbiome

## Abstract

*Staphylococcus capitis* is a Gram-positive bacterium that is part of the normal human flora, found in multiple anatomical sites. Here, we present the 2.5-Mbp draft genome of *S. capitis* O112, isolated from a cheek swab collected from a healthy female.

## ANNOUNCEMENT

*Staphylococcus capitis* frequently is a benign member of the human skin microflora, also found in other organs, for example, the urinary tract and gastrointestinal tract (1). It is an opportunistic pathogen and has been isolated from neonatal-unit-associated sepsis cases (2), prosthetic joint infections (3, 4), and prosthetic valve endocarditis cases (5). Antibiotic resistance is of mounting concern, and a recent comparative analysis of *S. capitis* genomes found that 48% were classified as multi-drug resistant, most notably those strains of the *S. capitis* subspecies *ureolyticus* (6). Here, we present the draft genome sequence of *S. capitis* O112, isolated from a cheek swab collected from a healthy female.

*S. capitis* O112 was isolated as part of an approved institutional review board (IRB) study (Loyola University Chicago #3603). The study was limited to asymptomatic, females (18–24 years old) who had not taken antibiotics in the prior 6 months; written consent was received. Cheek swabs were collected (BD BBL CultureSwab), diluted with 0.7% saline solution 1:100, spread on a Mannitol Salt (MS) agar plate, and incubated for 24 hours at 35°C with 5% CO_2_. A single colony was selected to inoculate 1 mL of MS media and incubated for 24 hours at 35°C with 5% CO_2_. This process of colony purification was carried out two times.

An MS plate was sent to SeqCenter (Pittsburgh, PA, USA) for sequencing. There, a loopful of colonies were selected and DNA was extracted using the ZymoBIOMICS DNA Kit. A library was prepared using the Illumina DNA Prep kit and IDT 10 bp unique dual indices. Sequencing was conducted using the Illumina NovaSeq 6000 platform, yielding 1,167,425 pairs of 150 bp reads. Read quality control and assembly were performed using the BV-BRC web tool (v3.33.16) (7). First reads were trimmed and normalized using TrimGalore (v0.6.5dev) and BBNorm (v October 19, 2017), respectively. The reads were then assembled using Unicycler v0.4.8 with two rounds of polishing *via* Pilon v1.23. The Prokaryotic Genome Annotation Pipeline (PGAP) v4.9 (8) generated the genome annotation. Unless otherwise noted, default parameters were used for all software tools.

The draft genome sequence is 2,474,921 bp long, assembled into 26 contigs with an *N*_50_ value of 594,170 bp, a GC content of 32.72%, and a coverage of 198.919×. Genome species designation was determined by the Similar Genome Finder service through BV-BRC; the closest reference strain to *S. capitis* O112 is *S. capitis* strain FDAARGOS_173 (Distance 0.011; Accession no. GCF_001471555.1). dDDH analysis *via* the type strain genome server (TYGS) (9) identified the most similar strain as *S. capitis* ssp. *urealyticus* strain DSM 6717 (dDDH_6_ = 94.9%; Accession no. GCF_002901925.1), thus suggesting O112 is a member of this subspecies. The genome annotation includes 2,429 genes. One resistance gene, *fusB* (fusidic acid), was identified using ResFinder (v4.1) (10). The PGAP annotation includes genes associated with fosfomycin resistance. Both have previously been noted as prevalent for the species (6).

## Data Availability

This whole-genome shotgun project has been deposited in GenBank under accession no. SAMN38470372. The genome assembly accession number is JAXOTT00000000. The raw sequencing reads have been deposited in the SRA under accession no. SRR26969012.
